# Influenza viruses circulation and the effectiveness of seasonal influenza vaccine in Romania during the season 2013-2014

**DOI:** 10.1186/1471-2334-14-S7-P84

**Published:** 2014-10-15

**Authors:** Daniela Pițigoi, Emilia Lupulescu, George Necula, Maria Elena Mihai, Carmen Maria Cherciu, Cristina Tecu, Viorel Alexandrescu

**Affiliations:** 1 Cantacuzino National Institute for Research and Development for Microbiology and Immunology, Bucharest, Romania; 2 Carol Davila University of Medicine and Pharmacy, Bucharest, Romania

## Background

Influenza is a serious disease, causing sickness in about 5-15% of the population every season, of which about up to 1-3% die every year in Europe. The epidemiological and virological surveillance of influenza provide information necessary to detect novel viruses with pandemic potential, to guide designing appropriate vaccines and prophylaxis. We aimed to investigate the profile of influenza viruses circulating in Romania during the season 2013-2014 and to estimate the effectiveness of the seasonal influenza vaccine.

## Methods

We tested all specimens collected from patients with influenza like illness (ILI) in the national surveillance system from week 40/2013 to week 20/2014. Influenza A/B positive specimens identified by molecular detection (RT-PCR) were further characterized. We used hemagglutination inhibition assay for antigenic characterization and chemiluminescence assay for the antiviral susceptibility testing. Subsequently we conducted nucleotide sequencing of hemagglutinin and neuraminidase genes and phylogenetic tree analyses. We estimated influenza VE using the test negative case-control study design, as 1-odds ratio of vaccination among ILI cases positive for influenza and ILI negative controls. The Cantacuzino Institute ethical committee approved the study. No personal data were transmitted with questionnaires and patients gave a written informed consent to be swabbed.

## Results

The influenza activity started in Romania in the first week of 2014, when more than 10% of samples tested were positive, reached the peak in week 10 and lasted until week 18/2014. We tested 709 specimens, and 291 cases were positive (60.8% influenza A(H3N2), 35.4% A(H1N1)pdm09, 3.8% influenza B). Fifty-seven influenza viruses were antigenically and/or genetically characterized. The isolates AH1N1 pdm09 and H3N2 corresponded with the viruses recommended by WHO for inclusion in the 2013/14 northern hemisphere seasonal influenza vaccine. Influenza B viruses belonged to B/Yamagata/16/88 lineage clade 3 represented by B/Stockholm/12/2011. All tested strains (46) demonstrated susceptibility to oseltamivir and zanamivir, except for influenza type B strain, whose IC50 value was at the upper limit of the baseline previous season. The crude IVE against any influenza (N=147) was 66% (95%CI: -47; 94) and the adjusted IVE for age group and week of swabbing was 68% (-95; 95).

## Conclusion

Influenza activity was lower compared to the last three seasons and the season 2013-2014 was characterized by a mixed virological picture, with domination of influenza A(H3N2). The vaccine effectiveness suggests a moderate protection, taking into account the small sample size and the low vaccination coverage (2.7%).

